# Non-native listener perceptual similarity ratings as a measure of L2 speech production

**DOI:** 10.1121/10.0037229

**Published:** 2025-07-24

**Authors:** Carissa A. Diantoro, Melissa A. Redford

**Affiliations:** Linguistics Department, University of Oregon, Eugene, Oregon 97403, USA carissad@uoregon.edu, redford@uoregon.edu

## Abstract

This study investigates the feasibility of using non-native listeners to assess non-native pronunciation of different target languages. Native English-speaking students studying university-level German and French reproduced German, French, and Indonesian sentences based on native-speaker models. Students not pursuing university-level language study did the same. Other native English-speaking students served as non-native listeners, rating the similarity of elicited sentences to native-speaker models. Similarity ratings were higher for language learners compared to non-learners across languages. Articulation rate and interval-based rhythm measures were strong predictors of similarity ratings. Results parallel findings from second-language acquisition studies, wherein native listeners typically evaluate L2 pronunciation.

## 1. Introduction

Speech produced by adult second language (L2) learners differs from speech produced by native speakers. Significant differences between L2 pronunciation of a language and native-like pronunciation can render a learner's speech difficult for others to understand (Bent *et al.*, 2007; Zielinski, 2006). Given this, the learner's best path for improving functional communication is to minimize the perceptual distance between L2 speech and native speech. To aid the L2 learner along this path, a distance metric between L2 and native speech is required. Yet, most research on L2 pronunciation relies on native listeners' accentedness ratings (Munro, 2017). Such ratings include information orthogonal to the L2 learner's goal of improving functional communication. The specific problem is that accentedness ratings are more strongly associated with the listener's subjective feeling of how easy or hard the speech is to process (i.e., comprehensibility or perceived listening effort) than to the listener's actual ability to understand L2 speech (i.e., intelligibility) (Huensch and Nagle, 2021; Munro and Derwing, 1995; Verbeke and Simon, 2023). Moreover, native listener ratings of accentedness and comprehensibility are influenced both by experience (Flege and Fletcher, 1992; Kang *et al.*, 2016) and by sociolinguistic biases (Baese-Berk *et al.*, 2020; Kutlu, 2023). The effect of these influences on accentedness ratings is orthogonal to helping learners improve functional communication; thus, new metrics are needed to characterize the distance between a learner's pronunciation and their L2 target. The current study addresses this need.

Despite the problems associated with native listeners' ratings of speech, our view is that a perceptually based measure of L2 pronunciation is preferable to an instrumental-acoustic one. After all, an effective instrumental-acoustic measure of L2 pronunciation requires selecting some subset of speech features that influence intelligibility and then weighting these appropriately according to their relative influence on speech perception. It is both more direct and more effective to instead obtain a holistic perceptual measure of pronunciation where the selection, weighting, and integration of features are done automatically by the human listener. Our proposed distance metric thus follows the inherent logic of the commonly used accentedness ratings: When native listeners rate the accentedness of L2 speech, they evaluate the given utterance against some imagined native speech version of that utterance. Our task makes this comparison explicit. We provide listeners with a target sentence produced by a native speaker plus the learners' repetition of this sentence. Listeners then directly rate the similarity of the two sentences. Critically, we recruited listeners who do not speak the target language to provide these ratings. The rationale for using non-native listeners follows from a broader literature on perceptual comparison (Medin *et al.*, 1993; Smith and Heise, 1992): Perceivers reference signal-based features of two stimuli when making a comparison if they lack conceptual knowledge relevant to making the comparison. Given this, non-native listener similarity ratings may provide the metric we need to characterize distance between a learner's pronunciation and their L2 target; that is, one that controls for the top-down influences of listener experience and sociolinguistic biases on L2 speech ratings.

In the current study, we assess the validity of using non-native listeners' perceptual similarity ratings of L2 speech to assess L2 pronunciation. We elicited French and German sentences from native English-speaking L2 learners of these languages. The learners were university students recruited from 200-level language classes (i.e., low-intermediate language learners). We also elicited French and German sentences from a control group of “non-learners”—that is, from a group of native-English speaking students who were not pursuing foreign language study at the university level and showed no intention of doing so. If perceptual similarity ratings are a valid measure of proficiency, then French and German sentences produced by low-intermediate learners of these languages should be rated as more similar to native French and German models of the sentences than sentences produced by non-learners.

Assuming that the similarity ratings provided by non-native listeners indeed distinguish learner pronunciation from non-learner pronunciation of a target language, we might also use similarity ratings to investigate second language speech acquisition more generally. In the current study, we do this by testing the hypothesis that French and German L2 learners have a learning advantage over non-learners when it comes to attempting a novel language, namely, Indonesian. The hypothesis is motivated by second-language acquisition study findings of a bilingual language-learning advantage over monolinguals (Antoniou *et al.*, 2015; Beach *et al.*, 2001; Kaushanskaya and Marian, 2009; Kopečková, 2018). By hypothesis, French and German learners will outperform non-learners in reproducing the sound patterns of Indonesian. Perceptual similarity ratings on Indonesian sentences should therefore be higher for L2 learners as a group compared to non-learners. Our experimental design controls for the possibility of L2 language transfer on production in the perceptually critical domain of rate and rhythm.

### 1.1 Influences of rate and rhythm

Second language acquisition researchers have long observed that a speaker's first language (L1) strongly influences their pronunciation of an L2 when the L2 is acquired after childhood. This phenomenon is known as language transfer (Flege, 1995; Li and Post, 2014; McAllister *et al.*, 2002; Odlin, 1989). It is also possible that the knowledge gained in L2 learning influences a speaker's pronunciation of additional languages (e.g., Beach *et al.*, 2001; Kehoe and Havy, 2019; Kopečková, 2016, 2018). Our selection of French, German, and Indonesian as target languages was designed to control for both types of transfer effects in the perceptually critical domains of rate and rhythm: English and German are considered stress-timed languages (Dauer, 1983), while French and Indonesian are considered syllable-timed languages (Browne, 1977; Dauer, 1983). By including both French and German learners as speakers in the study, we are able to distinguish learning effects from transfer effects in the pronunciation of target languages. For example, non-learners and L2 French learners will both benefit from English in the pronunciation of German; French learners may benefit from L2 transfer in the pronunciation of Indonesian, whereas German learners will be as constrained as non-learners by negative transfer from English.

Rhythm class differences are due to syllable structure, the presence/absence of lexical stress, and the presence/absence of vowel reduction (Dauer, 1983; Ramus *et al.*, 1999). These phonological features influence the relative duration of interleaved vocalic and consonantal intervals, hence the use of interval-based metrics to characterize L2 speech rhythm (e.g., Fuchs, 2023; Ordin and Polyanskaya, 2015; White and Mattys, 2007). They also influence the rate at which syllables are produced per second, hence the possibility of differentiating stress- and syllable-timed languages based on speech rate (Dellwo, 2008). Therefore, in the current study, we computed interval-based rate and rhythm metrics to test for the relative influence of these features on listener perceptual similarity ratings as a function of learner group.

Both rhythm and rate are well known to influence the perceived “accentedness” of speech (Anderson-Hsieh *et al.*, 1992; Derwing *et al.*, 1998; Field, 2005). Although prior research has shown minimal to no effects of language background on global L2 speech ratings (Mackay *et al.*, 2006; Major, 2007; Munro *et al.*, 2006), other work suggests that listeners may weight segmental features more highly than suprasegmental ones in their assessment of L2 speech when L2 speakers are proficient enough in the target language to roughly approximate native-like rhythm (Huensch and Nagle, 2021). If this same pattern holds for similarity ratings, then we would expect two-way interactions between learner group and rate/rhythm metrics in models of these ratings; that is, we would expect that similarity ratings will vary more strongly with rate and rhythm differences in non-learners' speech compared to L2 learners' speech.

## 2. Methods

### 2.1 Participants

This section reports only those participant characteristics directly relevant to the study aim. The supplementary material provide greater detail on participants characteristics (e.g., demographic information).

#### 2.1.1 Speakers: L2 learners and non-learners

A total of 30 speakers were recruited into the study and financially compensated for their participation. The non-learner group was 12 monolingual English speakers, which are those who were not pursuing foreign language study at the university level and showed no intention of doing so. The L2 learner groups were 12 English-speaking L2 French learners and 6 English-speaking L2 German learners. Non-learners were recruited by word-of-mouth and from introductory psychology classes at the University of Oregon. L2 learners were recruited from second-year language courses in French and German, respectively. By the time of their participation in the study, the L2 learning students had completed at most one 3-month term of second-year French or German study (i.e., French/German 201). Consistent with the low-intermediate language learning status defined by this level of study, L2 learners self-reported moderate proficiency in their language of study as measured on a scale from 1–7, with 1 being very low and 7 being native like. The average self-reported proficiency for the French learners was 4.21 (SD = 1.1); it was 4.17 (SD = 0.75) for the German learners.

#### 2.1.2 Non-native listeners

A total of 122 English-speaking students were recruited as listeners from the human-subjects pool maintained by the Department of Psychology at the University of Oregon. Data from two listeners were discarded due to zero variability in their similarity ratings across all stimuli. The remaining 120 non-native listeners ranged in age from 18 to 34 years old (mean = 19.4, SD = 1.9).

### 2.2 Procedure

#### 2.2.1 Sentence elicitation

The stimuli used for the elicitation task consisted of 5 sentences in French, German, and Indonesian drawn from textbooks of these languages (French: Consumer, 2012; German: Stocker, 2012; Indonesian: Oey and Davidsen, 2013). The sentences were controlled for number of syllables (*N* = 8–10) and syntactic complexity (consists of only one clause) (see supplementary material). A female native speaker of each language (*N* = 3) produced each of the 5 sentences in their respective languages. Their speech was recorded in a sound-attenuated room with a Shure SM81 standing microphone connected to a Marantz PMD660 digital recorder. Each sentence was repeated three times by the speaker. The second repetition of each sentence served as the target stimuli in the sentence elicitation task, which is described next.

All 30 L2 learners and non-learners were presented with pre-recorded versions of the sentences produced by a native speaker. Their task was to repeat each sentence as accurately as possible. Sentence elicitation was blocked by language. Speakers were told which language they were to produce before the start of each block. The order of the blocks was randomized across speakers. Each sentence was presented five times in random order within a language block. Speakers were asked to repeat the sentence twice upon hearing it, yielding a total of 10 repetitions per sentence. In total each speaker produced 50 sentence elicitations per language (5 sentences × 5 presentations × 2 repetitions). Sentences were initially recorded in a sound-attenuated room using a Shure SM81 standing microphone connected to a Marantz PMD660 digital recorder. Data collection transitioned to Zoom due to COVID-19 protocols. In the end, 8 non-learners speakers, 4 French learners, and 3 German learners were recorded using Zoom. Recording location (in-lab vs online) was included as a fixed effect in preliminary analyses. It was a non-significant effect in all models and so is not considered further.^1^

#### 2.2.2 Perceptual similarity rating

The rating experiment was conducted online, through Testable (www.testable.org). It was blocked by target language within listeners. The order of the blocks was counter-balanced across listeners. Perceptual similarity ratings were obtained on the middle 4 repetitions of each sentence in every target language to limit the number of sentences in the rating experiment from 4500 to 1800 (30 speakers × 5 sentences × 3 languages × 4 repetitions). Each of the 1800 sentences was paired with the native speaker model of the sentence. The model sentence always appeared first in the pair. The two sentences were separated by a 250-ms silent interval. The paired sentences for a particular language were randomized and divided into batches of 300 sentence pairs each. Listeners were randomly assigned one batch of paired sentences per language. Each batch was rated by a minimum of 20 listeners.

Listeners were informed that their task was to rate the acoustic-auditory similarity of paired sentence stimuli. They were told that the sentences were produced in languages other than English and that the first sentence in a pair of sentences was always produced by a native speaker of the target language and the second by a non-native speaker. They were not given any information about the target languages or learner/non-learner status of the speakers. The listeners' task was to rate the perceived distance between the first and second sentence in a pair on a 7-point Likert scale, anchored with the text “very far” for 1 and “close to native” for 7. For each language block, listeners first completed four practice trials before proceeding to rating the target sentences. Similarity ratings and response times were automatically recorded. Similarity ratings on trials with response times of less than 2000 ms or more than 9000 ms were excluded from the analyses following the procedure from previous studies (Gosling and Mason, 2015; Johnson, 2005).

### 2.3 Acoustic segmentation

All French, German, and Indonesian sentences that were rated by listeners were also acoustically segmented into pause-delimited utterances and then into vocalic and consonantal intervals using Praat (Boersma and Weenink, 2020). Vocalic and consonantal intervals were identified following Ramus *et al.* (1999) as stretches of all vocalic or all consonantal material. Glides were treated as consonants when prevocalic and as vowels when post-vocalic; all other sonorants were treated as consonants. For example, the phrase “next Tuesday on” would be segmented as /n//ɛ//kstj//u//zd//eiɔ//n/ (Ramus *et al.*, 1999, p. 272). Pauses were identified based on listening and silent gaps of any length in the signal. If a pause occurred after a stop consonant, the boundary was placed after the consonant release if a release was present; it was placed at vowel offset if a release was not present. In ambiguous cases, such as the silent gaps associated with stop closures, the silent interval had to be perceived as a pause and it had to exceed 200 ms to be identified as a pause. Once segmented, articulation rate was calculated per sentence repetition as the number of syllables per second minus the cumulative duration of pauses. The rhythm metrics calculated were percent vocalic duration of the sentence absent pauses (%V) and the standard deviation of vocalic interval durations (ΔV) and consonantal interval durations (ΔC). Syllable-timed languages (e.g., French and Indonesian) are reported to have higher %V and lower ΔV and ΔC than stress-timed languages (e.g., English and German) (see Ramus *et al.*, 1999).

### 2.4 Analyses and predictions

Perceptual similarity ratings were *z*-scored within listener and language block to normalize for different implicit rating scales across listeners and languages. Separate linear mixed-effects models were built for each target language (French, German, Indonesian). In a first set of analyses, learner group was entered as the sole fixed effect. Random intercepts for speaker and listeners' L2 experience were included.^2^ For the models of French and German sentences, learner group was a three-way fixed effect (non-learner, opposite L2 learner, L2 learner); for the model of Indonesian sentences, L2 French and L2 German learners were combined and so learner group was a two-way fixed effect (non-learner vs L2 learner). A second set of analyses was conducted to test for effects of rate and rhythm on similarity ratings as a function of learner group. Random intercepts for speaker and for listeners' L2 experience were also included.

All models were created with the lmer function within the lme4 package (Bates *et al.*, 2015) in R (RStudio Team, 2024). Model comparison was used to test for the fixed effects of learner group on similarity ratings in the first set of models and for the fixed effects of learner group and rate and rhythm on similarity ratings in the second set of models. Chi-squared statistic and corresponding p value are reported when the fixed effect of learner group was significant alone or in interaction with rate and rhythm. *Post hoc* comparisons tested for pairwise differences in similarity ratings on French and German sentences, where the fixed effect of learner group was a three-way variable. These comparisons were made using the emmeans function from the emmeans package (Lenth, 2022); alpha levels were adjusted using the Bonferroni method.

The predicted effects of group on similarity ratings were as follows: (1) Similarity ratings will be higher on French and German sentences produced by L2 learners of these languages compared to L2 learners of the opposite language and non-learners; (2) similarity ratings will be higher on Indonesian sentences produced by L2 learners of French and German than on those produced by non-learners. The predicted effects of rate and rhythm measures on ratings were as follows: (3) Similarity ratings will vary systematically with rate and rhythm measures in a manner consistent with target language expectations for these measures; and (4) similarity ratings will vary more strongly with rate and rhythm measures on sentence produced by non-learners and opposite-language learners compared to L2 learners, consistent with an effect of proficiency on the relative weighting of suprasegmental vs segmental features (Huensch and Nagle, 2021).

## 3. Results and Discussion

### 3.1 Overall group differences

**Fig. 1.**
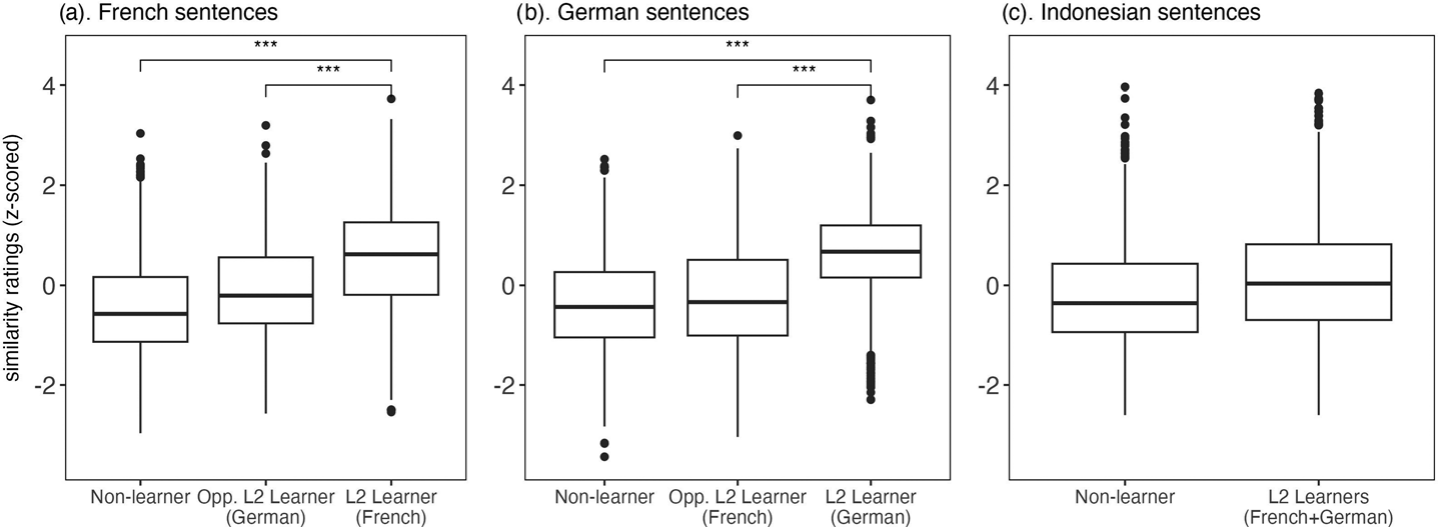
Normalized similarity ratings by learner group and language.

The effect of learner group on non-native listeners' similarity ratings of French and German sentences was significant (French: *χ*^2^(2) = 32.82, p < 0.001; German: *χ*^2^(2) = 22.65 p < 0.001). Figures 1(a) and 1(b) show that sentences produced by L2 learners of these languages were rated more highly than sentences produced both by non-learners and by the opposite L2 learners. The effect of learner group on similarity ratings of Indonesian sentences also approached significance (*χ*^2^(1) = 3.83, p = 0.05): As a group, sentences produced by French and German learners received somewhat higher similarity ratings than non-learners on the Indonesian sentences.

The similarity rating results on French and German sentences are on keeping with the expectation that the similarity ratings can be used to assess L2 speech proficiency: Pairwise comparisons indicated that French and German L2 learners received higher similarity ratings on sentence repetitions in their target language than either non-learners (French L2 vs non-learners: β = −0.94, SE = 0.20, *p* < 0.001; German L2 vs non-learners: β = −1.00, SE = 0.19, p < 0.001) or than the opposite L2 learners (French L2 vs opposite L2: β = −0.61, SE = 0.25, p < 0.05; German L2 vs opposite L2: β = −0.90, SE = 0.19, p < 0.001). Given that the L2 learners in question were recruited early in the second year of their language course work, the systematic effect of group on similarity ratings suggests that these provide a sensitive measure of L2 speech proficiency.

The similarity rating results on Indonesian sentences provide weak support for the prediction of an L2 language learning advantage on the reproduction of entirely novel language. Although sentences produced by low-intermediate L2 learners receive somewhat higher perceptual similarity ratings than those produced by non-learners [Fig. 1(c)], pairwise comparisons across the three learning groups indicated no significant differences. Relatedly, pairwise comparisons indicated no difference in perceptual similarity ratings on French and German sentences produced by opposite language learners compared to those produced by non-learners, despite a trend in this direction for French sentences (German L2 vs non-learner: β = −0.33, SE = 0.25, p = 0.55). Together, the results suggest either that the similarity ratings may not be sensitive enough to detect subtle differences in first attempts producing a novel language or that low-intermediate L2 learners may not benefit from an L2 language learning advantage. The latter possibility is at least as likely as the former since the language learning advantage has only been reported in studies with advanced L2 learners and functional bilinguals (e.g., Antoniou *et al.*, 2015; Beach *et al.*, 2001; Kaushanskaya and Marian, 2009).

### 3.2 Influences from rate and rhythm

Model analyses that included rate and rhythm metrics as predictors suggest the influence of these features on perceptual similarity ratings regardless of target language: Similarity ratings on French sentences varied systematically with all rate and rhythm measures (rate: *χ*^2^(1) = 610.50, p < 0.001; %V: *χ*^2^(1) = 43.96, p < 0.001; ΔV: *χ*^2^(1) = 6.31, p < 0.05; ΔC: *χ*^2^(1) = 5.26, p < 0.05); ratings on German sentences varied systematically with all measures except ΔV (rate: *χ*^2^(1) = 182.08, p < 0.001; %V: *χ*^2^(1) = 30.69, p < 0.001; ΔC: *χ*^2^(1) = 7.83, p < 0.01); and ratings on Indonesian sentences varied systematically with all measures except %V (rate: *χ*^2^(1) = 392.66, p < 0.001; ΔV: *χ*^2^(1) = 60.83, p < 0.001; ΔC: *χ*^2^(1) = 20.84, p < 0.001). Models that included only rate and rhythm metrics as fixed effects explained 23% of the variance in perceptual similarity ratings on French sentences (R^2^m = 0.229; R^2^c = 0.420), 22% of the variance in similarity ratings on German sentence (R^2^m = 0.223; R^2^c = 0.335), and 14% of the variance on Indonesian sentences (R^2^m = 0.141; R^2^c = 0.253).

**Fig. 2.**
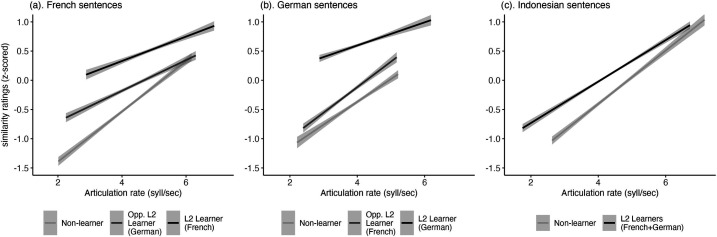
Interaction between articulation rate and learner group on similarity ratings by language.

**Fig. 3.**
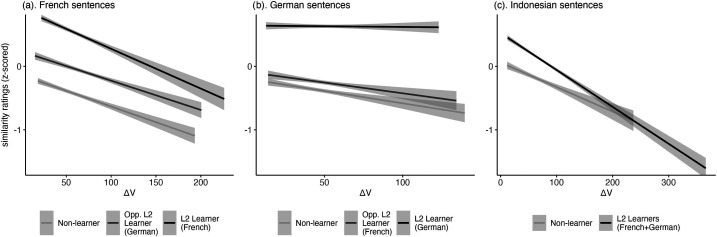
Interaction between standard deviation of ΔV (units in milliseconds) and learner group on similarity ratings by target language.

As expected, rate and rhythm measures also interacted with learner group in models on similarity ratings: The effect of %V and ΔV on similarity ratings of paired French sentences varied systematically with learner group (%V: *χ*^2^(2) = 41.76, p < 0.001; ΔV: *χ*^2^(2) = 57, p < 0.001); the effect of rate, %V and ΔC on similarity ratings of paired German sentences varied systematically with learner group (rate: *χ*^2^(2) = 11.29, p < 0.01, %V: *χ*^2^(2) = 8.64, p < 0.05; ΔC: *χ*^2^(2) = 8.77, p < 0.005); and, the effect of ΔV and ΔC on similarity ratings of paired Indonesian sentences varied systematically with learner group (ΔV: *χ*^2^(1) = 7.74, p < 0.01; ΔC: *χ*^2^(1) = 4.04, p < 0.05). Figures 2 and 3 show the group by measure interaction for rate and ΔV. Figures showing the interaction effect with ΔC and %V are included in the supplementary material.

The relationship between articulation rate non-native listeners' perceptual similarity ratings was consistent with our expectations based on second-language acquisition studies. As in our results (Fig. 2), previous studies have found that faster speaking rates are associated with more native-like pronunciation of a target language (Kang, 2010; Munro and Derwing, 1998).

Unlike the effect of articulation rate on similarity ratings, the effect of different rhythm metrics on similarity ratings ran contrary to our expectations from language specificity. Regardless of whether the language was stress- or syllable-timed, especially high values of %V, ΔV, and ΔC predicted lower similarity ratings. The interaction between these metrics and learner group was also not consistent with our expectations from proficiency. For example, contrary to expectations from proficiency, metrics like %V and ΔV were as likely to predict similarity ratings of French sentences produced by low-intermediate French learners as to predict similarity ratings of these sentences produced by non-learners (see Fig. 3).

## 4. Conclusion

The findings from this study show that non-native listeners' ratings on the perceptual similarity of paired non-native and native sentences provide a distance metric that can be used to assess L2 pronunciation proficiency. The results clearly show that the metric is sensitive enough to distinguish speech produced by low-intermediate L2 learners of a target language from that produced by non-learners of the language. The metric may also be sensitive enough to pursue research on theoretical questions within the domain of second language acquisition, such as the extent to which speaking two languages enhances one's ability to learn a novel third language. Further, the results indicate that non-native listeners attend to articulation rate and other rhythm-related measures when rating the similarity of a non-native sentence to a native model sentence, if not always in the direction expected based on the association of the rhythm metrics with language rhythm classes. The current set of results on the acoustic correlates of perceived similarity parallels findings from accentedness and proficiency ratings studies of second language acquisition (e.g., Kang, 2010; Munro and Derwing, 1998). Although we did not examine effects or correlates of segmental articulation on similarity ratings in the present study, the much higher ratings on French and German sentences produced by L2 learners compared to those produced by non-learners together with expected interactions between group and rate/rhythm predictors strongly suggest that our non-native listeners also attended to segmental features in their similarity ratings of paired native and non-native sentences.

## Supplementary Material

See supplementary material for the list of sentences used for elicitation, figures of the relationship between other rhythm measurements and perceptual similarity ratings, and tables with all model results.

## Data Availability

The aggregated data and R script are available on the Open Science Framework at https://osf.io/vjp9s/.

## Footnotes

^1^
Recording location was added as a fixed effect in the models of similarity ratings. We compared mixed effect models that contained this effect to models that included only group and, in the case of the second set of analyses, the rate and rhythm measures. Model comparisons showed no significant effect of recording location. We therefore proceeded without this variable in the reported analyses.

^2^
Fifty-eight listeners reported having some L2 learning experience. Six of these 58 listeners had studied French. We ran models with and without these 6 listeners and obtained the same results. Therefore, we proceeded to include all listeners in the model. None reported having studied German or Indonesian.
